# Overcoming the limitations of immunotherapy in pancreatic ductal adenocarcinoma: Combining radiotherapy and metabolic targeting therapy

**DOI:** 10.7150/jca.92502

**Published:** 2024-02-12

**Authors:** Han Zhang, Wenjin Xu, Haitao Zhu, Xuelian Chen, Hsiang-i Tsai

**Affiliations:** 1 Institute of Medical Imaging and Artificial Intelligence, Jiangsu University, Zhenjiang, China.; 2 Department of Medical Imaging, The Affiliated Hospital of Jiangsu University, Zhenjiang, China.; 3 Department of Radiology, Affiliated Kunshan Hospital of Jiangsu University, Kunshan, Jiangsu, China.

**Keywords:** pancreatic ductal adenocarcinoma, immunotherapy, radiotherapy, metabolic reprogramming, tumor microenvironment

## Abstract

As a novel anticancer therapy, immunotherapy has demonstrated robust efficacy against a few solid tumors but poor efficacy against pancreatic ductal adenocarcinoma (PDAC). This poor outcome is primarily attributable to the intrinsic cancer cell resistance and T-cell exhaustion, which is also the reason for the failure of conventional therapy. The present review summarizes the current PDAC immunotherapy avenues and the underlying resistance mechanisms. Then, the review discusses synergistic combination therapies, such as radiotherapy (RT) and metabolic targeting. Research suggests that RT boosts the antigen of PDAC, which facilitates the anti-tumor immune cell infiltration and exerts function. Metabolic reprogramming contributes to restoring the exhausted T cell function. The current review will help in tailoring combination regimens to enhance the efficacy of immunotherapy. In addition, it will help provide new approaches to address the limitations of the immunosuppressive tumor microenvironment (TME) by examining the relationship among immunotherapy, RT, and metabolism targeting therapy in PDAC.

## 1. Introduction

Pancreatic ductal adenocarcinoma (PDAC) is the third leading cause of mortality from cancer among men and women, with approximately 23,930 deaths from 30,920 diagnoses globally in 2023. In addition, the condition is associated with a high incidence of solid tumor^1^, ^2^. By 2030, PDAC is expected to be the second leading cause of cancer-related deaths^3^. Currently, the 5-year survival rate of patients after surgery is only 20%-25%. This is because most patients are diagnosed in the late stages of the disease. As a result, surgical resection and conventional adjuvant treatment (chemotherapy and thermotherapy) improve survival in only a small percentage of patients.

In recent years, immunotherapy has demonstrated favorable results in the treatment of melanoma, renal cancer, and uroepithelial carcinoma. This treatment includes the use of immune checkpoint inhibitors (ICIs) that by inhibit immune checkpoint molecules such as programmed cell death protein-1 (PD-1), programmed cell necrosis protein ligand-1 (PD-L1), and cytotoxic T-lymphocyte-associated antigen 4 (CTLA-4). However, translational clinical studies assessing PDAC have not revealed favorable results. PDAC is characterized by an immunosuppressive tumor microenvironment (TME) that is comprised of cancer-associated fibroblasts (CAFs) and its derived extracellular matrix as well as immunosuppressive cells such as regulatory T cells (Tregs), tumor-associated macrophages (TAMs), myeloid-derived suppressor cells (MDSCs), and neutrophils (NETs); this TME is the most significant contributor to anticancer treatment failures^4^. Immunosuppression is primarily mediated by the release of immunosuppressive soluble mediators, such as interleukin 10 (IL-10) and transforming growth factor-β (TGF-β); the increased expression of immunosuppressive receptors such as PD-1 and CTLA-4 on antitumor immune cells; and the inhibition of essential metabolic substrates such as tryptophan and arginine by immune cells^5^. Owing to such alterations, cancer cells can escape surveillance by the immune system. In addition, cancer cells recruit several immunosuppressive cells that secrete immunosuppressive factors to inhibit the immune response, induce immune escape, and promote tumorigenesis. Currently, various synergistic immunotherapies have been developed. In addition, radiotherapy (RT) induces an immune response that enhances the immunogenicity of PDAC. Thus, combining RT with immunotherapy may improve the immunosuppressive microenvironment of PDAC. In addition, targeting tumor metabolism may enhance the efficacy of immunotherapy via the metabolic reprogramming of TME, thereby improving the function of immune cells.

RT is an important treatment modality for intermediate to PDAC. Conventional RT aims to maximize the radiation dose to the tumor site while minimizing damage to the normal site^6^. Although clinical studies have highlighted the importance of RT as a treatment modality, the modulatory role of the immune system in cancer treatment has been neglected^7^. A recent study has found that RT can induce an *in situ* vaccination effect by causing cancer cell death and activating systemic immune response^8^, ^9^. The most prominent example is the abscopal effect, in which RT administered at one site may result in tumor regression at distal nonirradiated areas^10^, ^11^. In RT-sensitive breast cancer model, RT enhances antitumor immunity by augmenting the secretion of pro-inflammatory cytokines, tumor-associated antigens, and chemokines, which contributes to the establishment of a peritumoral proimmunogenic milieu^12^. In contrast to PDAC, RT causes the release of cytokines that mediate the polarization or recruitment of immunosuppressive cells, hence strengthening the TME's immunosuppressive qualities. These findings lay the groundwork for rationalizing immunotherapy in combination with RT for cancer treatment. In certain instances, RT and immunotherapy have demonstrated a synergistic antitumor effect and improved the prognosis of patients with PDAC^13^. Nonetheless, the immunosuppressive TME of PDAC has become the most significant barrier to immunotherapy and RT combination therapy.

In addition to the fact that RT can enhance the efficacy of immunotherapy by increasing the immunogenicity of PDAC, metabolic modulation to improve the function of immune cells can additionally improve the clinical outcome of immunotherapy. In PDAC cells, metabolism is characterized by a high dependence on glycolysis and a reduced dependence on oxidative phosphorylation (OXPHOS). These metabolic alterations significantly contribute to PDAC cell proliferation and metastasis. The aberrant phenotype and metabolic modifications of PDAC facilitate an immunosuppressive TME that promotes cancer cell proliferation^14^. In addition, the increased consumption of glucose in the TME restricts the availability of nutrients to immune cells in the TME, compromising the normal function of immune cells^15^. Simultaneously, cytokines and other factors secreted by PDAC cells induce phenotypic changes and, ultimately, metabolic reprogramming of immune cells^16^. PDAC is the type of cancer induced by metabolic alterations and immune suppression. Given the importance of immune cell metabolic profiling in PDAC, this review aims to examine the effects of RT on immune cells and their metabolic changes in the TME of PDAC to provide novel insights into the development of unique immunotherapy combination regimens.

## 2. Immunotherapy in PDAC

Although immunotherapy has demonstrated efficacy in numerous malignancies^17^, ^18^, it is ineffective in PDAC owing to numerous mechanisms. One factor is the dense stroma in the TME of PDAC which creates a physical barrier that impedes immunotherapy drug delivery. In addition, numerous cytokines, receptors, and metabolites produced by the TME play a significant role in reducing antigen presentation and inhibiting immune cell proliferation.

### 2.1 Clinical investigation of immunotherapy for PDAC

#### 2.1.1 ICIs

ICIs enhance the anti-tumor immune response by targeting T cells, thus boosting the immune response against tumors. Although blocking immune checkpoints using ICIs has demonstrated good efficacy in certain solid tumors, such as melanoma, renal cell carcinoma, and non-small cell lung cancer, it has little efficacy in PDAC^19^, ^20^. Currently, ICIs primarily target PD-1, PD-L1, and CTLA-4. Similar to PD-1, immune molecules such as TIGIT, TIM3, and LAG3 are inhibitory receptors. CD73 is a primary enzyme implicated in the synthesis of extracellular adenosine, a potent immunosuppressive, pro-angiogenic, and pro-tumor factor that accumulates in tumors. Vista is a novel checkpoint ligand homologous to PD-1/PD-L1 that inhibits T-cell activation. Although these immunosuppressive pathways are widely expressed in PDAC, the suppression of these immune pathways alone is ineffective.

PD-1 inhibits T lymphocytes and other immune cells by binding to its ligand PD-L1. However, the inhibition of both PD-1 and its ligand activates T cells and enhances the immune response^21^, ^22^. Reportedly, the binding of PD-1 to its ligands, PD-L1 or PD-L2, inhibits T-cell effectors, antagonizes T-cell receptor signaling, and inhibits transcription^23^-^25^. Pembrolizumab and nivolumab are PD-1 checkpoint inhibitors that are used to treat PDAC in a state of high satellite instability (MSI-H)^26^. In a follow-up study of a single-arm, phase 2 trial (NCT01876511) enrolling 86 patients with PDAC, durable responses (objective response rate [ORR] 53% [95% confidence interval: 42%-64%]) were observed, with 21% of patients achieving a complete response^27^. Nevertheless, the recent KEYNOTE-158 trial revealed that only 4 of 22 patients achieved partial remission or complete response^28^. However, the proportion of PDAC patients with MSI-H was between 0.8% and 2%, indicating that PD-1 inhibition did not achieve the desired efficacy for most patients with PDAC. In another study of a subcutaneous PDAC mouse model, anti-PD-1/PD-L1 therapy significantly reduced tumor volume^29^. However, the clinical efficacy of anti-PD-1/PD-L1 therapy alone^19^, ^30^ or in combination^31^ has demonstrated poor efficacy, with an ORR of only 0%-3%, median progression-free of 1.5 months, and overall survival of 3.1 months. These findings demonstrate that immune checkpoint blockade is ineffective for the treatment of PDAC for various reasons.

CTLA-4 acts on tumor tissues and peripheral circulation, whereas PD-1/PD-L1 acts only on the tumor. Recent research suggests that PD-1/PD-L1 recruitment affects peripheral T cells and tumor tissue^32^. Specifically, PD-1 immune checkpoint inhibition primarily affects CD8^+^ T cells. In contrast, CTLA-4 induces an indirect effect on CD8^+^ T cells by regulating CD4^+^ T cells and effector cells.

#### 2.1.2 Vaccines

In addition to ICIs, vaccines play a significant role in cancer immunotherapy, modulating immune-related pathways, and inducing or enhancing antitumor immune responses^33^.

Antigenic vaccines are categorized into neoantigen and tumor-associated antigenic vaccines. Neoantigen vaccines are specific vaccines that target neoantigens. Neoantigens are non-autologous proteins specific to cancer cells and are produced by nonsynonymous mutations^34^. Since neoantigens are only expressed by cancer cells, they are unique targets for tumor immunotherapy as they stimulate an immune response by activating CD4^+^ and CD8^+^ T cells^35^. To date, clinical trials evaluating various neoantigen vaccines, such as peptide vaccines, have demonstrated high efficacy in patients with PDAC^36^. However, the efficacy of these vaccines varies significantly among patients, making it crucial to individualize their study^37^. Tumor-associated antigenic vaccines consist primarily of RAS peptides, mucin 1 (MUC1) peptides, and telomerase peptides^38^-^40^. Kras mutations are the most prevalent mutations (95%) in PDAC^41^. Using a combination of mutated Kras peptides and granulocyte-macrophage colony-stimulating factor (GM-CSF), some patients demonstrated a specific immune response to KRAS mutations^42^, ^43^. MUC1 is a highly glycosylated, high-molecular-weight protein that is widely distributed on the surface of cancer cells and plays an essential role in cancer development and metastasis. Yamamoto et al. found the level of circulating anti-MUC1-IgG antibodies was increased in patients with PDAC who received a combination of MUC1 peptide and an adjuvant^44^. Studies have demonstrated that telomerase activity is inhibited in normal human tissues and is high in tumors; therefore, its activity has been elevated in all solid tumor types.

Cellular vaccines include allogeneic cellular vaccines, autologous cellular vaccines, and dendritic vaccines. Allogeneic vaccines are produced using cancer cells and are then administered to elicit an immune response in another patient. GM-CSF gene transduced autologous tumor vaccine (GVAX) is an allogenic vaccine that has been extensively studied in PDAC. GVAX is essentially a group of PDAC cell lines (including CG8020/CG2505) that have been genetically modified to secrete GM-CSF for immune regulation. The study by Laheru et al. treated patients with PDAC using GVAX alone or in combination with cyclophosphamide. Treatment with GVAX induced mesothelin (MSLN)-specific T-cell responses in a few patients. Moreover, GVAX, either alone or in combination with cyclophosphamide, exhibited minimal treatment-related toxicity, indicating the good safety of the vaccine^45^. In a mouse model of PDAC, Saung et al. demonstrated that GVAX treatment alone increased colony-stimulating factor 1-receptor (CSF-1R) expression in lymphoid polymers and was associated with a lower survival rate. In contrast, the combination of GVAX + anti-PD-1 + anti-CSF-1R increased the infiltration of PD-1^+^CD137^+^CD8^+^, PD-1^+^CD137^+^CD4^+^, and PD-1^+^OX40^+^CD4^+^ T cells within the tumor. Moreover, PD-1^+^CD137^+^CD8^+^ T cells induced high levels of interferon-γ (80%-90%) in response to CD3/CD28 activation using stimulation beads. Thus, the combination of anti-PD-1 antibodies, GVAX, and anti-CSF-1R antibodies may be an effective approach for the management of PDAC^46^. These findings indicate that irrespective of its resistance and poor efficacy, adding RT may enhance the effectiveness of antitumor vaccine immunotherapy.

#### 2.1.3 Adoptive cell therapy

In adoptive cell therapy, tumor regression is induced by acquiring and expanding a patient's tumor-specific T cells *in vitro* and then transferring them to the patient. This treatment has demonstrated favorable clinical outcomes in malignant hematologic diseases^47^ but not PDAC. Chimeric antigen receptor T-cell (CAR-T) therapy is the most rapidly evolving clinical therapy. A study has found that MUC1-pulsed dendritic cells (DCs) activate T lymphocytes in patients' peripheral blood mononuclear cells. One patient with multiple lung metastases receiving this treatment experienced complete remission, and five patients with stable disease were among the twenty patients tested. The average length of survival was 9.8 months^48^. Reportedly, Carcinoembryonic Antigen (CEA) and MUC1 are overexpressed on the surface of PDAC cells and therefore are the most promising targets for CAR-T^49^, ^50^. In a mouse model of PDAC, treatment with CAR-T targeting CEA and MUC1 demonstrated a reduction in tumor size^51^, ^52^. Although adoptive cell therapy is a novel and promising field, it is associated with a few limitations. The most challenging aspect of CAR-T research is the selection of antigens, and the most recent CAR-T studies target tumor-associated antigens rather than tumor-specific antigens. Tumor-associated antigens lack specificity and exhibit variable or heterogeneous expression on tumor cells, which is associated with a significant risk of off-target toxicity. As with other immunotherapies, improving the efficacy of pericyte therapy alone in PDAC is difficult.

### 2.2 Immunosuppression TME, the cause of immunotherapy's inefficacy

Despite the promising therapeutic outcomes observed with immunotherapies such as ICIs, vaccines, and adoptive cell therapy in the treatment of renal cell carcinoma, non-small cell lung cancer, and melanoma, the present prognosis regarding PDAC remains unsatisfactory. Furthermore, optimistic outcomes of the majority of immunotherapeutic modalities have been observed exclusively in clinical trials involving PDAC patients with the MSI-H subtype. Regrettably, the MSI-H subtype comprises a mere 0-2% of all PDAC subtypes^53^, indicating a deficient proportion of PDAC patients who exhibit sensitivity to immunotherapy. Other factors impede immunotherapy^54^, apart from the fact that the majority of PDAC patients are in advanced stages at the moment of consultation. For instance, PDAC complex oncogenes override adaptive T-cell immunity to promote immunosuppression in early PDAC^55^. Furthermore, PDAC metastasis could hide signals of medication efficacy by creating additional immunological barriers along the cancer-immune axis^56^. Lastly, tumors depend on an immunosuppressed TME to evade the immune system. Therefore, in order to investigate the intrinsic mechanism of PDAC immunosuppression and devise combination therapies with immunotherapy, we must begin with the intricate immunosuppressive TME of PDAC.

#### 2.2.1 CAFs and Immunosuppression

A critical determinant in determining the prognosis of PDAC is fibrosis^57^, ^58^. Approximately 70% of the tumor tissue is comprised of the stromal component, and CAFs are significant constituent cells of the stroma. CAFs facilitate fibrosis while demonstrating an array of immune influences that promote tumor suppression^59^.

Extensive connective tissue proliferation results from CAFs-mediated fibrosis in PDAC, which induces vascular collapse and generates high interstitial fluid pressures, thereby restricting the diffusion and perfusion of immunotherapeutic agents and small molecules. Paolo et al. significantly enhanced overall survival by targeting hyaluronic acid, normalizing interstitial fluid pressures, and combining it with chemotherapy to treat connective tissue hyperplasia. Hypoxic TMEs and the collapse of small blood vessels within the PDAC result in CD81 T cell suppression mediated by Tregs^60^. CAFs are also capable of facilitating immunosuppression through their interaction with cytotoxic lymphocytes (CTLs).C-X-C Motif Chemokine Ligand 12 (CXCL12) and activation of focal adhesion kinase (FAK) by CAFs restrict the migration of CTLs to stromal compartments, thereby impeding T cell initiation^61^. Jiang et al.^62^ established that FAK activity is a crucial regulator of fibrosis and immunosuppressive TME in PDAC and that the FAK inhibitor VS-4718 substantially slowed tumor progression and increased survival. In a murine model of PDAC, Feig et al. significantly increased CD8^+^ T-cells by combining anti-PD-L1 therapy with a CXCL12 antagonist, thereby delaying the progression of the tumor. Moreover, prostaglandin E1, TGF-, Indoleamine 2,3-dioxygenase (IDO), arginase, and IL-10, which CAFs all secrete, have the potential to inhibit CTL function^63^. Lastly, by interacting with these immunosuppressive cells, inflammatory factors secreted by CAFs (IL-6, GM-CSF, and vascular endothelial growth factor) can promote their differentiation and reprogramming.

#### 2.2.2 Immunosuppressive cells—the key actors in the TME

Approximately 50% of the TME of PDAC is comprised of immunosuppressive cells TAMs, MDSCs, and Tregs. By promoting the polarization and recruitment of these immune cells, the immunosuppressive features of the TME of PDAC are enhanced. The predominant variety of TAMs in the TME of PDAC is M2 (pro-tumourigenic phenotype). The Dectin1/galectin-9 axis directly induces apoptosis of T cells^64^ and PD-L1^65^ expression in TAMs. IL-10, which is maintained in functional Treg populations, promotes Th2 development and is produced by M2 TAMs^66^. By increasing IL-12 expression in DCs, inhibition of IL-10 enhanced the infiltration and chemotherapeutic effectiveness of CTLs. TAMs are also capable of inhibiting CTLs via the production of arginase-1^66^. MDSCs are highly efficient producers of nitrogen species and reactive oxygen, which disrupt the activity of TCRs and IL-2^66^. MDSCs induce proliferative arrest of antigen-activated T cells by depleting micronutrients via arginase-1-dependent consumption and L-cysteine sequestration, thereby downregulating the T-cell receptor^67^. Beyond their capacity to disrupt TCRs, MDSCs are also capable of inducing T-cell apoptosis and promoting the activation and proliferation of Tregs. More T-cells infiltrated the tumor stroma subsequent to the genetic deletion of CXCR2, a chemokine receptor that is predominantly present on Gr-MDSCs^68^. By inhibiting MDSCs via CXCR2 blockade in a genetically engineered mouse model of PDAC, fibrosis was reduced, MDSCs within the TME were diminished, and ICB acted synergistically with these effects^69^. CD4 helper T cells exhibit a tumor-promoting Th2 phenotype and are abundant in the TME in comparison to CD8 T cells^70^. While Treg density is lower in frequency compared to Th2 cells, it exhibits an upward trend as the disease advances. It has been associated with lymph node metastases and unfavorable survival outcomes^71^, ^72^. PDAC cells secrete a slew of cytokines linked to Treg migration and accumulation, including IL-10, TGF-B, and CCL5^73^. These immunosuppressive T cells block CD86 and CD40, among other anticancer immunologic actions.CD86 and CD40 are necessary for the activation of CD81 T cells and the development of local immunological suppression^74^, ^75^. By eliminating Tregs in a PDAC mouse model, DCs were able to induce a CD81-dependent, potent antitumor immune response^76^. However, in a clinically relevant genetic model of PDAC, Treg depletion had no effect on CD8 T-cell recruitment, implying that Treg removal alone is insufficient to restore productive T-cell immunity^77^.

## 3. Effect of RT on TME

**Figure 1:**
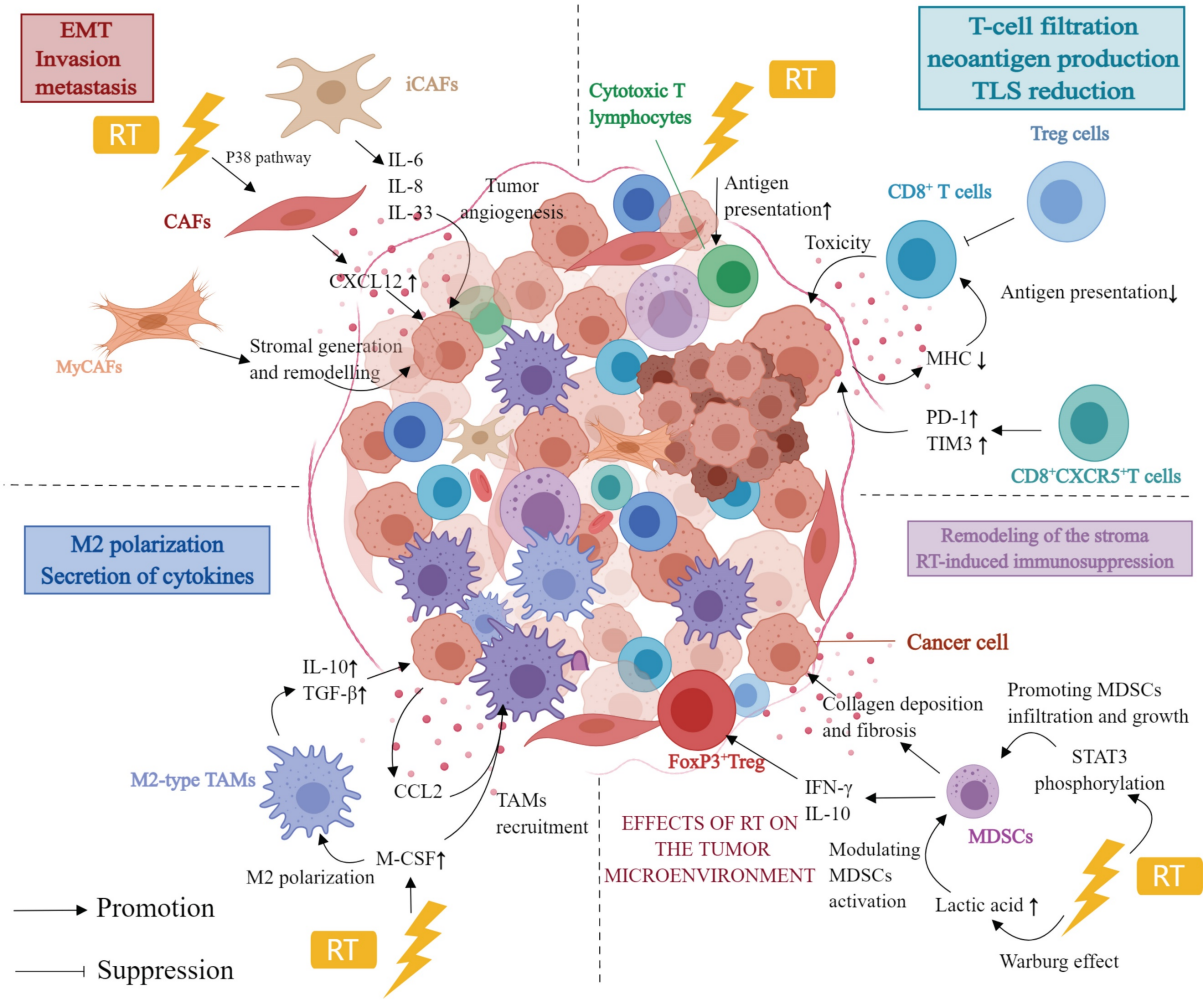
The effect of RT on immune crosstalk in the TME of PDAC. Abbreviation: TME: tumor microenvironment; PDAC: pancreatic ductal adenocarcinoma; RT: radiotherapy; EMT: epithelial-mesenchymal transition; CAF: cancer-associated fibroblasts; iCAFs: inflammatory cancer-associated fibroblasts; myCAFs: myofibroblastic cancer-associated fibroblasts; MDSCs: myeloid-derived suppressor cells; M-CSF: macrophage colony-stimulating factor. Created with MedPeer (https://www.medpeer.cn/; accessed on 28 April 2023).

In PDAC, the TME is highly complex and dynamic. Specifically, the stroma is a critical component of the PDAC microenvironment, consisting primarily of immune cells, fibroblasts, and blood vessels. The complex TME in PDAC renders immunotherapy alone ineffective^78^; therefore, evaluating different combination therapies is critical for improving the prognosis of patients with PDAC. Notably, a recent study has demonstrated that RT affects the TME (Figure 1). Owing to its immunosuppressive TME, PDAC is classified as a “cold” tumor, and immunotherapy alone has a suboptimal effect. Therefore, it has been hypothesized that combining immunotherapy with RT can overcome immunotherapy tolerance observed in PDAC. The combination treatment can directly cause DNA damage to PDAC cells as well as modulate immune responses, such as antigen presentation and T-cell activation^79^. Thus, immunotherapy combined with RT is likely to emerge as a promising treatment option to transform a “cold” tumor into a “hot” one.

### 3.1 RT induces immune responses in the TME of PDAC

RT may induce a systemic immune response, the most representative example of which is the abscopal effect. However, the abscopal effect observed is a rare event, and there appears to be no therapeutic value of this effect in clinical practice. With the increasing understanding of immunotherapy, it was realized that RT also induces a significant immunostimulatory effect, and combining immunotherapy with RT may enhance the abscopal effect^80^, ^81^. The abscopal effect is closely related to RT-induced systemic immune response, which involves reprogramming the TME and immune cell infiltration. Although international guidelines certify RT as a treatment modality, the exact mechanism, dose, duration, and efficacy in PDAC remain to be studied^82^.

### 3.2 CAFs and RT

The stroma in PDAC tumors comprises the vast majority of the tumor and consists of fibroblasts, blood vessels, and extracellular matrix. CAFs, mesoderm-derived heterogeneous cells, are a significant component of the TME and are associated with cancer at all stages of progression^83^. CAFs in PDAC are classified into three major subtypes: inflammatory CAFs, myofibroblastic CAFs, and antigen-presenting CAFs. Inflammatory CAFs can be triggered by IL-1 and further secrete various cytokines and chemokines, such as IL-6, IL-8, and IL-33. These cytokines and chemokines regulate other immune cells in the TME, including TAMs, MDSCs and DCs, to create an immunosuppressive TME. Initially, IL-6 produced by CAF was found to promote monocyte bias towards M2 polarization^84^. Numerous pro-inflammatory cytokines (CXCL2, GM-CSF, CCL2, CSF-1) have been implicated in the recruitment of MDSCs and the polarization of M2-type TAMs, both of which contribute to the formation of an immunosuppressive TME^85^-^87^, as determined by CAF proteomics research. In recent years, et al. demonstrated that STAT3-mediated IL-6 production by CAF of PDAC can induce the MDSC phenotype in monocyte precursors. TSLP, which CAF additionally secretes, can polarize DCs through polarization, thereby promoting fibrosis and an immunosuppressive Th2 phenotype^88^. Myofibroblastic CAFs located near cancer cells have anti-tumor and myogenic functions related to stromal generation and remodeling. Because different CAF subtypes have different anti-tumor and pro-tumor effects, blindly targeting CAFs does not immediately improve efficacy.

RT can impact tumor progression and treatment sensitivity. CAFs, unlike cancer cells, are believed to be highly resistant to RT. CAFs survive and undergo senescence despite receiving high doses of RT. However, RT induces long-term DNA damage in CAFs, which inhibits their proliferation^89^. Ohuchida et al. demonstrated in a co-culture system of PDAC cells and CAFs. Compared to CAFs without RT treatment, co-culture of CAFs after RT (5 Gy or 10 Gy) with PDAC cells can significantly enhance the invasive ability of PDAC cells^90^. According to Al-Assar et al., PDAC cells increase the radio-resistance of PDAC cells by releasing TFG-β and other factors, thereby promoting epithelial-mesenchymal cell transformation^91^. Furthermore, RT-treated CAFs promote CXCL12 secretion via the upregulation of the P38 pathway, which promotes invasion, metastasis, and epithelial-mesenchymal cell transformation of pancreas cancer cells^92^. To summarize, the function of RT in treating PDAC is far more complex than that identified in studies. As previously stated, the effect of CAFs on the response of cancer cells to radiation varies based on CAF subtypes. Moreover, the anti-tumor or pro-tumor effects of CAFs are affected by cancer cell type, RT pattern, and dose^93^.

### 3.3 Immune cells and reactive T Cells

The immunological microenvironment of PDAC has various immune cell types and subtypes, including TIL, TAM, MDSCs, and tumor-associated NETs. These cell types can interact with one another as well as crosstalk with TME, resulting in pro-tumor or tumor-suppressive outcomes^94^, ^95^. Understanding the effect of RT on different types of immune cells is therefore critical for both understanding the TME in PDAC and developing a rational therapeutic strategy.

#### 3.3.1 TILs

Most TILs in the TME of PDAC support cancer invasion and progression. TILs include various subgroups of cytotoxic (CD8^+^) T cells, Treg cells, and natural killer cells^96^. Studies have reported that RT promotes T-cell filtration by inducing DNA damage as well as functions as an *in situ* vaccine, inducing neoantigen production and eliciting a tumor-specific immune response^97^. Reportedly, Balachandran et al. found that rather than the number of neoantigens, neoantigen quality was a biomarker for immunogenic tumors. Thus, when combining RT and immunotherapy, the combination producing the highest quality neoantigens must be screened. As a result, stereotactic body RT (SBRT) appears more appropriate than CRT for treating PDAC when combined with ICIs^98^. SBRT has been shown to enhance antigen delivery and stimulate the number of cytotoxic T lymphocytes in a mouse model of PDAC^99^. Moreover, patients treated with SBRT have demonstrated a significant reduction in the distribution of CD8^+^/CD4^+^ T cells within the tumor as well as a substantial reduction in the number of perivascular CD8^+^ T cells^100^. It has been reported that as precancerous lesions transform into PDAC, tertiary lymphoid structures (TLS) accumulate immunosuppressive cells. SBRT treatment has been found to reduce immunosuppressive cell accumulation in TLS and decrease TLS area and number in the PDAC tissue^100^.

#### 3.3.2 TAMs

TAMs are immune cells derived from the bone marrow that are recruited into the TME and promote the initiation and progression of PDAC^101^. TAMs are negatively correlated with the prognosis of patients with PDAC. There are two major subtypes of macrophages: M1 (antitumor) and M2 (protumor). M1 exerts its antitumor effects via the secretion of pro-inflammatory cytokines, whereas M2 promotes tumor angiogenesis and invasion^102^. In contrast, the M2 phenotype dominates the TME of PDAC, releasing multiple immunosuppressive factors, including IL-10, Chemokine Ligand 2(CCL2), and TGF-β^103^, ^104^. The significance of TAMs in immunosuppression, angiogenesis, stromal remodeling, and cancer cell invasion has been demonstrated in preclinical PDAC mouse models^105^-^107^. Moreover, preclinical studies have demonstrated that depletion and reprogramming to target TAMs have yielded positive results, but it is challenging to attain efficacy in clinical studies^104^.

Most TAMs in the TME of PDAC are M2-polarized, a characteristic linked to immunosuppression and pro-tumor proliferation. Seifert et al. established a PDAC mouse model and found that RT induced a higher proportion of immunosuppressive M2 TAMs^108^, which may inhibit T cell-mediated antitumor responses. Moreover, RT induced the secretion of macrophage colony-stimulating factor (M-CSF), which resulted in the recruitment of TAMs and their M2 polarization. Treatment with M-CSF inhibitors prevented RT from modifying the proportion of M2 macrophages while delaying tumor progression^108^. Notably, the effect of RT on the recruitment of TAMs and their polarization of M2 phenotype could only be observed at early time points after RT, and M-CSF expression returned to the baseline levels after some time^108^. This indicates that the inflammatory stimulus induced by RT for recruiting and programming TAM may diminish over time. M2 TAMs secrete more immunosuppressive cytokines, such as IL-10 and TGF-β, following RT^109^, ^110^.

Immunosuppressive factors and chemokine receptors released by M2 TAMs, including CCL2 and CCR2/CCR5, are also regulated by RT and are linked to RT resistance. Kalbasi et al.^111^ have found that RT promotes the synthesis of CCL2 in PDAC cells and induces the recruitment of TAMs in the TME, which may lead to RT resistance. In vivo experiments in PDAC mouse models have revealed that RT increased TAMs, inflammatory monocytes/macrophages 3 days after treatment. However, treatment with CCL2 antagonists inhibited the recruitment of macrophages and monocytes, restoring the sensitivity to RT and chemotherapy. Additionally, CCL2 knockdown in PDAC cells restores radiosensitivity. Contrary to popular belief, treatment with CCL2 inhibitors without RT is not effective against PDAC. A study by Wang et al. has demonstrated that CCR2 and CCR5 are associated with TAMs infiltration in the tumor; however, targeting CCR2 or CCR5 alone does not sensitize PDAC tumors to immunosuppression in the absence of T-cell initiation mechanisms^112^. The study determined that the combination of anti-PD-1 immunosuppressant, CCR2/CCR5 antagonists and RT (3 × 8 Gy) was found to inhibit the infiltration of M2-type TAMs, Tregs and MDSCs, and this combination had better anti-tumor effects.

#### 3.3.3 MDSCs

MDSCs can trigger FoxP3^+^ Treg production by secreting interferons and IL-10, thereby maintaining TME immunosuppression^113^. The granulocyte MDSC (G-MDSC) is the most prevalent subtype of MDSCs, accounting for roughly 80% of all MDSCs. It induces post-translational modifications in T-cell receptors via the STAT3-TANs pathway. The other subtype, monocyte MDSCs, accounts for approximately 20% of all MDSCs and inhibits T-cell responses while inducing T-cell apoptosis via the STAT1/NO axis^114^, ^115^.

Using a PDAC mouse model and human PDAC samples, Oweida et al. found that RT (8 Gy) increased the frequency of G-MDSCs and NETs. In addition, they demonstrated that RT-induced STAT3 phosphorylation was associated with MDSC infiltration and growth in PDAC. RT combined with STAT3 inhibition decreased collagen deposition and fibrosis, which resulted in remodeling of the stroma in PDAC TME and eventually reversing RT-induced immunosuppression^110^. Specifically, RT has been shown to increase MDSC pro-tumorigenic action. Specifically, RT induces the Warburg effect, which increases lactate secretion, modulates MDSC activation, and induces MDSCs to adopt an immunosuppressive phenotype^109^. However, another study revealed that RT (5 × 6 Gy) did not significantly alter MDSC cell populations in a PDAC mouse model^116^. Thus, further studies are required to evaluate the effect of RT at various irradiation doses on immune cells to determine the best treatment modality for PDAC.

## 4. Metabolic targeting: A breakthrough in PDAC therapy

Despite several advancements in the combination of RT and immunotherapy over the past few years, the combination has not been extensively used in clinical treatment, indicating the need for further studies for treating PDAC. It is believed that KRAS mutations and inactivating mutations in the oncogenes TP53, SMAD4, and CDKN2A are essential for cancer progression and refractoriness in PDAC^117^. As mentioned previously, the poor efficacy of combination therapy has also been linked to an immunosuppressive TME and extensive connective tissue proliferation^118^. Moreover, it has been discovered that cancer metabolism plays a significant role in limiting the therapeutic effect. Specifically, in recent years, reprogramming of cancer cell metabolism has reemerged as a prominent aspect of anticancer treatment^119^, ^120^. It is well known that cancer cells reprogram numerous metabolic pathways to mediate their growth, division, and survival. Recent findings suggest that metabolic alterations can promote the development of PDAC via epigenetic alterations^121^, ^122^. Further evidence has suggested that the metabolism of PDAC is closely related to RT tolerance and immunosuppression^123^, ^124^. By classifying patients with PDAC into distinct metabolic subgroups, namely quiescent, glycolytic, cholesterolemia, and mixed, it is possible to predict the prognosis and potentially feasible therapeutic approaches for the subgroups^125^, ^126^. Thus, analyzing the metabolic profile of PDAC offers emergent, viable treatment options^126^, ^127^, and treatments targeting metabolism may represent a breakthrough for the combination treatment of RT and immunotherapy.

### 4.1 Metabolic reprogramming in PDAC TME

The TME of PDAC comprises various components, such as cancer cells, stromal cells, immune cells, and extracellular matrix, and elucidating the metabolic crosstalk between these components^54^, ^128^ is essential for identifying effective therapeutic options. It is well known that cancer cells are characterized by a malnourished and hypoxic state as they are enveloped by a proliferating collagen network. This collagen network provides proline to cancer cells, thus promoting cancer cell survival and metabolism in the absence of adequate nutrition^129^. In addition, these proliferating connective tissues promote glycolysis via irregular shear stress, which results in the production of reactive oxygen species (ROS) and the upregulation of the PI3K/AKT signaling pathway^130^. In addition, cancer cell glycolysis results in the production of lactate, which must be eliminated without OXPHOS to maintain normal metabolism. Dovmark et al. have hypothesized that connexin-43 (Cx43) channels are essential for dissipating lactate anions from glycolytic PDAC cells and for transporting excess lactate from these cells in the core of the tumor to the periphery, which they act as a substrate for OXPHOS in the well-perfused, normal cells. Furthermore, Cx43 is also thought to be a novel target for influencing metabolite processing in connection-coupled tumors^131^. G-protein-coupled receptor 81(GPR81), a Gi-coupled receptor present on PDAC cells, promotes the expression of monocarboxylase transporters and CD147 and can detect lactate in the TME. Activated GPR81 can upregulate the levels of peroxisome proliferator-activated receptor gamma coactivator-1 and boost mitochondrial biogenesis and respiration. Together, lactate uptake and utilization increased, crucial for the growth of PDAC cells in the TME under low-glucose conditions following metabolic remodeling^132^. Hui et al. discovered that during fasting, the contribution of circulating lactate to TCA cycle intermediates exceeds that of glucose; moreover, lactate is the primary TCA substrate in most tissues and tumors^133^.

### 4.2 Metabolic reprogramming in CAFs and PSCs

**Figure 2:**
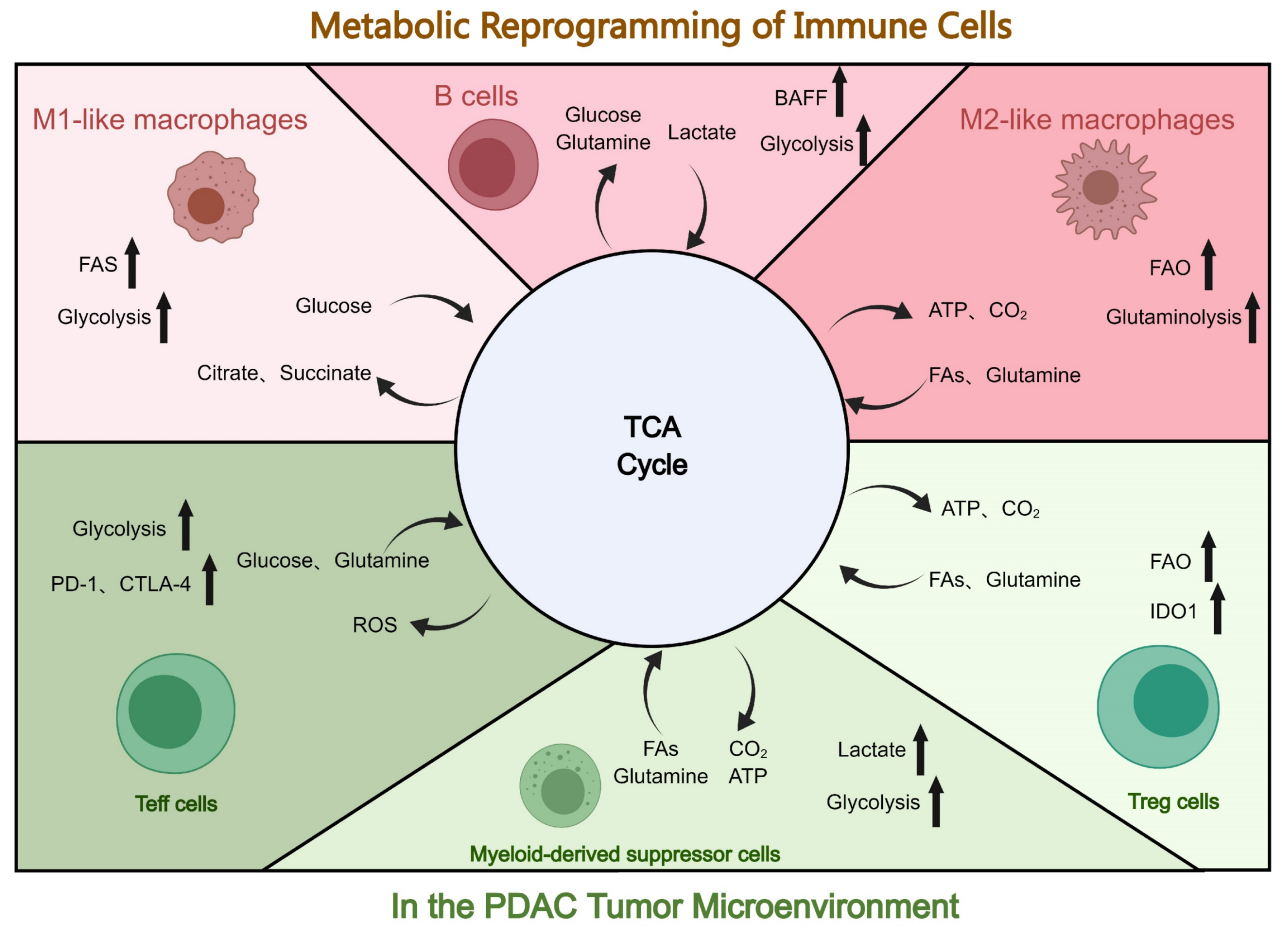
Reprogramming of immune cells in the tumor microenvironment of PDAC. Presented are metabolic changes in immune stromal cells (macrophages, T cells, B cells, and MDSCs). Created with MedPeer (https://www.medpeer.cn/; accessed on 28 October 2023).

Aerobic glycolysis, known as the Warburg effect, is a characteristic metabolic feature of cancer cells. CAFs, the major component of stromal cells, are stimulated by neighboring cancer cells and exhibit aerobic glycolysis while secreting a variety of metabolites, including lactate and pyruvate. It is believed that OXPHOS has been abandoned partially in CAFs. These metabolites produced by the CAFs' aerobic glycolysis are taken up by adjacent cancer cells, and this crosstalk is known as the reverse Warburg effect, which has received a great deal of attention in recent years^134^, ^135^. Shan et al. demonstrated that CAFs in PDAC exhibited a similar reverse Warburg effect. CAFs secreted more lactate in a co-culture model with PDAC cells, whereas PDAC cells promoted invasion and migration^136^. Besides, studies have found that CAFs release exosomes to supply the TME with metabolites, including amino acids, lipids, and TCA cycle intermediates, which are essential for the growth of cancer cells in the nutrient-deficient TME (Figure 2). Zhao et al. demonstrated that PDAC cells ingested CAFs derived exosomes may exhibit metabolism reprogramming, namely increased glycolysis and decreased OXPHOS^137^. The majority of CAFs originate from pancreatic stellate cells and can synthesize autophagy-derived alanine as an alternative carbon source when stimulated by cancer cells. These PSCs can be activated by PDAC cell-derived KRAS (G12D) signaling, which regulates cancer cell proliferation and apoptosis, increases mitochondrial capacity via the IGF1R/AXL-AKT axis, and modifies metabolism^138^. Bin et al. have identified that pancreatic stellate cells secrete the PSC-paracrine hepatocyte growth factor (HGF) via the HGF/c-MET/YAP/HIF-1 signaling, which promotes glycolysis in PDAC cells^139^. Moreover, saturated and monounsaturated fatty acids appear to have contradictory effects on fibrosis and PSC activation^140^. A combination of LipidemTM, an emulsion rich in omega-3 fatty acids, and gemcitabine substantially reduces PSC proliferation and inhibits PDAC cell invasion^141^.

### 4.3 Metabolic reprogramming of immune cells in the TME of PDAC

#### 4.3.1 Metabolic reprogramming of T cells

T cells can be categorized into natural killer cells, Tregs, C toxic T cells, memory T cells, Th cells, and gamma delta (γδ) T cells^96^, ^142^. These subpopulations of T cells demonstrate significant disparities in the metabolic profiles. Tregs, for instance, rely on fatty acid oxidation (FAO), whereas CD4^+^ Th cells and CD8^+^ Teff cells rely on glycolysis. In PDAC, these metabolic pathways regulate T-cell differentiation and immune responses against cancer cells^143^. Moreover, the concentration of amino acids in the TME affects the function of both cancer cells and activated T cells. Compared with adjacent nontumor tissues, glutamine is the amino acid most consumed by the PDAC cells^144^. In addition, glutamine fuels the TCA cycle, which produces nucleotides and proteins^145^. Studies have shown that the restriction of glutamine and leucine inhibits Th1 and Th17 differentiation but does not affect Treg differentiation^146^. Another metabolic alteration seen in PDAC cells is FAO. CD4^+^ T cells and PDAC cancer cells must upregulate the very-long-chain acyl-CoA dehydrogenase (VLCAD) enzyme to acclimatize to the lipid-rich microenvironment of the tumor. In contrast, CD8^+^ T cells selectively downregulate VLCAD, resulting in the accumulation of both long-chain fatty acids (LCFAs) and very LCFAs, which leads to lipotoxicity. This progressive accumulation of specific LCFAs in pancreatic CD8^+^ T cells impairs mitochondrial function, which induces transcriptional reprogramming of lipid metabolism pathways and ultimately decreases CD8^+^ T cell lipolysis^147^. T cells, like PDAC cells, are highly dependent on glucose as a nutrient and energy source. Moreover, the clinical use of PD-1, CTLA-4, and PD-L1 antibodies has been demonstrated to restore glucose levels in the TME. This restoration of glucose levels can activate T cells, stimulate the production of interferon-gamma, and initiate glycolysis^16^.

#### 4.3.2 Metabolic reprogramming of TAMs

Macrophages are an integral component of the immunosuppressive TME and participate in metabolic reprogramming. The function and phenotype of macrophages may be influenced by cellular metabolites. Compared to normal macrophages, TAMs possess a unique glycolytic profile that facilitates PDAC metastasis and angiogenesis, as demonstrated in metabolic flux assays^148^. Ye et al. have demonstrated that TAMs stimulate glycolysis in PDAC cells via a paracrine pathway. Moreover, lactate in the TME induces the polarization of TAMs towards the M2 phenotype, further suppressing immunity^149^. Metavert, as an inhibitor of glycogen synthase kinase 3 beta and histone deacetylases, can normalize glucose metabolism in PDAC cells and facilitate the conversion of oncogenic M2 TAMs to the anticancer M1 phenotype in a mouse model^150^. Moreover, M1 macrophages can inhibit mitochondrial TCA cycling, improve glycolytic metabolism, and produce ATP to sustain their phagocytosis^151^. Alternatively, lipid metabolism also plays a crucial role in macrophage activation and function regulation. Moreover, lipid metabolism enables macrophages to perform phagocytosis and secrete more cytokines more efficiently. Increased free fatty acids and lipoprotein absorption notably exacerbates the inflammatory response^152^.

#### 4.3.3 Metabolic reprogramming of MDSCs

MDSCs primarily utilize glucose, lipid, and amino acid metabolism pathways. MDSCs in the TME have a high glycolysis rate, and increased glycolysis significantly increases the levels of lactic acid, owing to which MDSCs consistently exert immunosuppressive activity. Additionally, increased glycolysis protects MDSCs from apoptosis, which reduces ROS production. In the hypoxic and nutrient-deficient TME, modulating MDSC function and glucose metabolism via HIF-1α results in the alteration of the tumor's hypoxic response. As a regulator of energy metabolism, AMPK has some effect on MDSCs and glycolysis. Tumor-invasive MDSCs acquire ATP via the crucial step of FAO activation. In contrast to polymorphonuclear MDSCs, which rely more on glycolysis and OXPHOS, mononuclear MDSCs accept ATP primarily through FAO activation. In addition, glutamine catabolism is essential in promoting MDSC maturation^153^, ^154^. In conclusion, the therapeutic approach for PDAC may be optimized by regulating MDSC metabolism.

#### 4.3.4 Metabolic reprogramming of NETs

The presence of numerous NETs in the TME of PDAC suggests that the function and number of NETs play an essential role in tumor development^155^. NETs participate in additional metabolic pathways, including glycolysis, glutamine catabolism, the TCA cycle, OXPHOS, the pentose phosphate pathway, and mitochondrial FAO. During the early phases of ROS production, NETs are primarily dependent on glycolysis, as opposed to the later stages, in which the cells are more dependent on mitochondrial FAO^156^. It is believed that for NETs, the primary metabolic substrate is glucose, which is metabolized via glycolysis and the pentose phosphate pathway. In tumor-associated NETs, metabolic alterations result from blood insufficiency, which causes nutrient deficiencies and local cytokine accumulation in the TME. The pentose phosphate pathway contributes nicotinamide adenine dinucleotide phosphate (NADPH) to the process by which NETs perform their biological functions. Reportedly, autophagy is activated in the nutrient-deficient TME to maintain metabolism. Inhibition of the essential autophagy gene Atg7 increases mitochondrial oxidation and glycolysis. Providing mitochondria with pyruvate and fatty acids promotes the formation of *Atg7*-deficient NETs and increases glycolysis and ATP production. Additionally, cells can use fatty acids and glutamine oxidized by tumor-associated NETs to synthesize additional ATP for tumorigenesis^157^. Furthermore, under limited glucose conditions, mitochondria produce ROS via NADPH oxidase and maintain intracellular NADPH levels. Studies also show that immature c-Kit^+^ NET subpopulations modulate mitochondrial oxidative metabolism^158^. In conclusion, the numerous metabolic patterns of NETs provide several novel strategies for the treatment of PDAC.

## 5. Combining immunotherapy: A clinical investigation

### 5.1 Immunotherapy combined with RT

#### 5.1.1 ICI combined with RT

In recent years, immunotherapy, particularly ICI combined with RT, has emerged as a new research focus^79^, ^159^ owing to the substantial immunomodulatory effects of RT. ICI combined with RT has achieved excellent efficacy in some solid tumors, such as non-small cell lung cancer and melanoma^160^-^162^; however, its efficacy in PDAC is suboptimal^163^, primarily due to the unique and complex immunosuppressive TME. Using an *in situ* homozygous PDAC mouse model, Fujiwara et al. assessed the efficacy of RT combined with PD-1 blockade and Indoleamine 2,3-dioxygenase; treatment. Of all combinations, low-grade RT (8 Gy × 3 times for 3 days) combined with PD-1 blockade significantly improved survival, resulted in the best systemic interferon-γ response; and induced the highest local expression of immune-activating genes (Cd28 and Icos)^164^. Azad et al. constructed KPC and Pan02 homozygous transplantation mouse models and found that high (12, 5 × 3. 20Gy) but not low (6,5 × 2Gy) RT doses combined with anti-PD-L1 significantly delayed tumor progression. Moreover, the combination of anti-PD-L1 and RT was associated with increased infiltration of CD45^+^CD8^+^ T cells and decreased infiltration of CD11b^+^Gr1^+^ myeloid cells^165^. In addition, the combination of RT and anti-PD-L1 increased T-cell activation markers (CD69, CD44, and FasL) as well as CD8: Treg ratios^165^. Using the UN-KC6141 mouse model of in situ PDAC, Lee et al. demonstrated that ablative RT combined with anti-PD-L1 significantly improved mouse survival. Specifically, 67% of mice in the combination group survived longer than 30 days after tumor inoculation; in contrast, the median survival time in the control group was 16.5 days. These findings imply that in combination with anti-PD-L1 treatment, ablative RT is more effective than conventional graded RT for recruiting T cells^166^. The ataxia telangiectasia-mutated gene (ATM) is the apical kinase that plays a role in mediating RT-induced DNA damage response. It has been shown that silencing ATM increases PD-L1 expression, thereby increasing the sensitivity of PDAC tumors to PD-L1-blocking antibodies. Using a subcutaneous PDAC mouse model, Zhang et al. have demonstrated that ATM inhibition improves the therapeutic efficacy of anti-PD-L1, which is further improved in combination with RT (8 Gy), resulting in enhanced tumor immunogenicity. This demonstrates the therapeutic potential of combining anti-PD-L1 and RT with ATM inhibition to treat PDAC^167^. In a recent phase 2 trial (NCT02704156), postoperative patients with locally recurrent PDAC received SBRT at doses ranging from 35 to 40 Gy. The findings revealed that increased doses of SBRT may improve the progression-free survival of pembrolizumab (anti-PD-1) and trametinib (MEK inhibitor) with gemcitabine; however, no improvement in the overall survival was noted^168^. A phase I study compared the safety of durvalumab (anti-PD-L1) and SBRT in patients with metastatic PDAC. Only two of the 39 patients evaluated for efficacy demonstrated partial responses, for an overall efficacy rate of 5.1%. Notably, no dose-limiting toxicity was observed, and lymphocytopenia was the most common adverse event. Thus, the safety profile of ICI combined with RT is acceptable for treating PDAC^169^. In a non-randomized, single-arm phase II trial (NCT03104439), RT was found to significantly increase the effectiveness of ICIs. The combination group using RT (3 × 8 Gy) with nivolumab (anti-PD-1) and ipilimumab (anti-CTLA-4) demonstrated a significantly higher disease control rate than the other treatment groups^170^. In an open-label, phase 2 randomized controlled study, the median overall survival (mOS) of patients in the SBRT + pembrolizumab + trametinib group was 24.9 months, whereas it was 22.4 months in the SBRT + gemcitabine group. Thus, SBRT combined with pembrolizumab and trametinib may be a novel treatment option for postoperative patients with PDAC. However, a phase III trial is needed to confirm these findings^171^. The postoperative pathology report of a patient with advanced PDAC preoperatively treated with RT and pembrolizumab (anti-PD-1) demonstrated a nearly complete pathologic response^172^. In contrast, an additional phase I trial combining low-split RT and pembrolizumab demonstrated that four patients with PDAC presented with progressive disease, with all RT target lesions metastasizing in the liver rather than the pancreas^173^.

CTLA-4 is a receptor that inhibits T lymphocyte activation; therefore, inhibiting CTLA-4 activates T lymphocytes^174^. In a phase 2 CheckPAC study (NCT02866383), patients with metastatic PDAC who were treated with nivolumab + ipilimumab (CTLA-4 blocker) + 15 Gy SBRT achieved an ORR of 37.2% (24.0-52.1) compared with those treated with nivolumab alone who achieved an ORR of 17.1% (8.0-30.6)^175^. Thus, ICI in combination with RT (SBRT) to treat patients with metastatic PDAC has implications for future research. The precise function of SBRT in the treatment remains to be determined.

Nonetheless, the majority of phase 1 and phase 2 clinical trials have failed to demonstrate efficacy in most patients with PDAC. However, combining immunosuppressive agents with RT or chemotherapy is crucial in the treatment of patients with prostate cancer. Data from the recent clinical studies combining ICI and RT or chemotherapy in patients with PDAC are listed in Table 1.

#### 5.1.2 Vaccine combined with RT

Algenpantucel-L is a vaccine comprising PDAC cells expressing the -1,3-galactosyltransferase gene. In a phase II clinical trial conducted by Hardacre, 70 patients with PDAC were treated with RT and Algenpantucel-L. After a median of 21 months of follow-up, this study yielded encouraging results, with a 12-month disease-free survival rate of 62% and an overall 12-month survival rate of 86%^176^. However, algenpantucel-L immunotherapy did not improve survival in patients with resectable or locally advanced unresectable PDAC at the treatment margin^177^. GVAX is a transgenic vaccine containing GM-CSF developed for the treatment of PDAC. In a phase 2 study involving 60 patients with PDAC, a combination of GVAX + RT resulted in a median disease-free survival of 17.3 months and a median survival of 24.8 months. Compared with the survival data on cancer resection, overall survival was higher when immunotherapy combined with RT, and patients tolerated the immunotherapy well^178^.

### 5.2 Immunotherapy in conjunction with metabolic targeting therapy

#### 5.2.1 Combination of ICIs and metabolic targeting therapy

ICIs have poor therapeutic efficacy in PDAC, whether used alone or in combination with RT^75^. This is primarily due to the complex TME of PDAC. An essential immune checkpoint receptor in activated immune cells is PD-1, which also promotes the proliferation of Tregs and has enhanced immunosuppressive properties. The binding of PD-1 to PD-L1 or PD-L2 induces inhibitory signals. Reportedly, the highly conserved C-terminal tyrosine of PD-1 is associated with Src homology region 2 domain-containing phosphatase-1 (SHP-1) and Src homology region 2 domain-containing phosphatase-2 (SHP-2) in both human and mouse transcripts. The interaction between T-cell receptor signaling and PD-1 induces the phosphorylation of the cytoplasmic tyrosine structural domain, which recruits SHP-2 to the C-terminal tyrosine.

**Figure 3:**
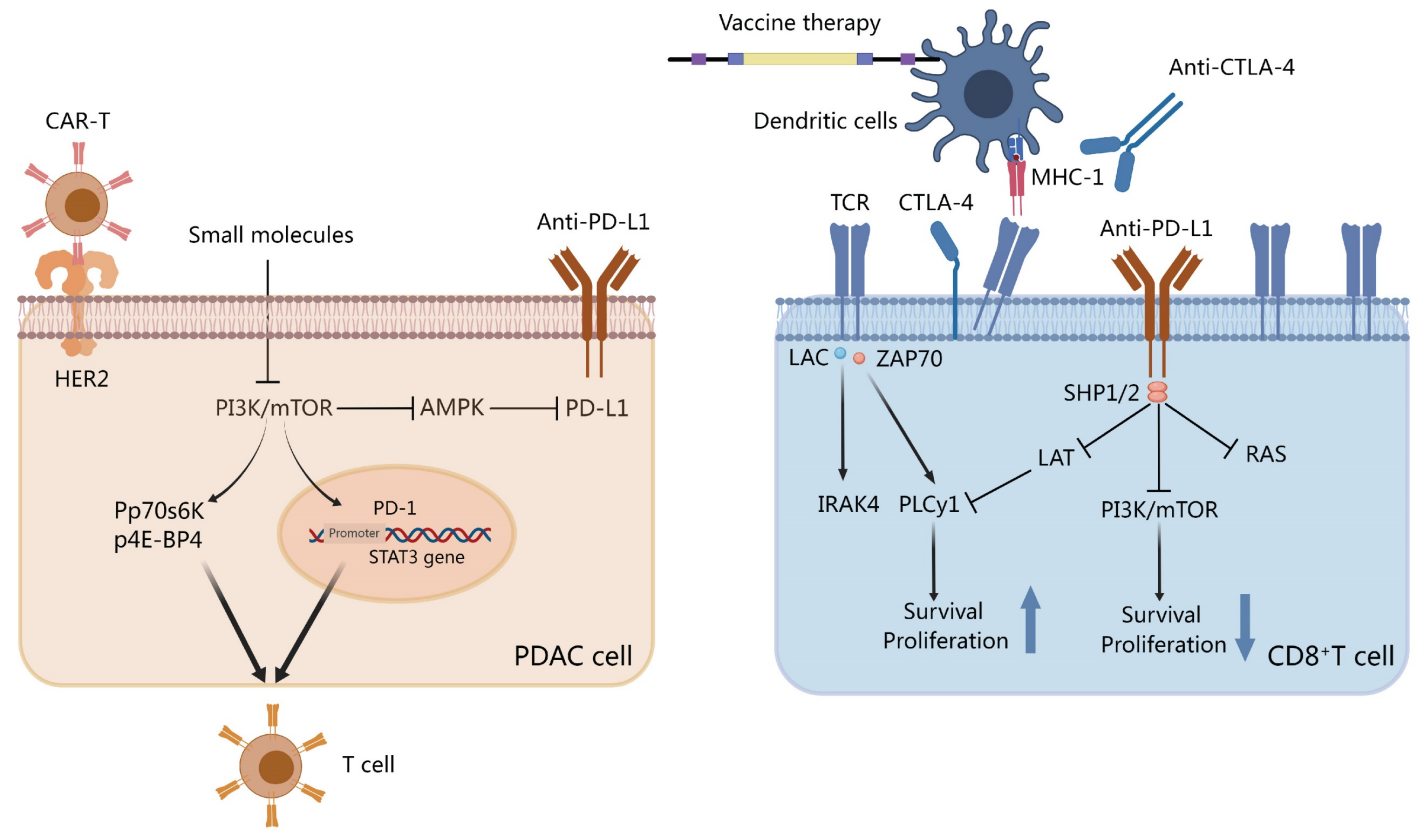
Small molecules and checkpoint inhibitors on Th cells and pancreatic ductal adenocarcinoma (PDAC). Programmed death-ligand 1 (PD-L1)/programmed cell death protein-1 (PD-1) sends stimulatory signals to T cells and inhibits PI3K activation. Small molecules inhibit the PI3K/mTOR pathway to inhibit PDL-1 and signal transducer and activator of transcription 3 (STAT-3) transcription. The activation of p-P70S6K and p-4E-BP1 affects Th cells via the mTOR pathway. Chimeric antigen receptor-T (CAR-T) cells target the HER2-specific PDAC receptor. Anti-CTLA-4, vaccine therapy, or both disrupt the interaction between dendritic cell T-cell receptor and MHC-1. Created with MedPeer (https://www.medpeer.cn/; accessed on 28 October 2023).

Consequently, SHP-2 dephosphorylates the Ras and PI3K-AKT pathways, inhibiting subsequent signaling (Figure 3). PI3K-AKT signaling inhibition decreases T-cell proliferation and cytokine production^179^. Moreover, PD-1 and PD-L1 may inhibit T-cell metabolism by inhibiting aerobic glycolysis as well as the PI3K/AKT/mTOR signaling pathway^180^. CTLA-4, another immune checkpoint receptor, can inhibit glucose uptake in cytotoxic T cells by binding to CD80/CD86, thereby downregulating the PI3K/AKT pathway. Unsurprisingly, it has been demonstrated that combination therapy with ICIs and metabolic targeting slightly enhances the efficacy of immunotherapy. Preclinical studies using murine melanoma models, anti-PD1 or anti-CTLA4 with glutaminase inhibitors, and other metabolic modulators have demonstrated that modulating the metabolism can increase the efficacy of PD-1/PD-L1 therapy^181^. Specifically, DAC cells are characterized by GLUT-1 receptor activation and an increase in lactate accumulation. In human PDAC samples, this increase in GLUT-1 expression was associated with increased PD-1 plus T cell density. These findings convey that both cancer and immune cells can reduce the efficacy of immunotherapy by activating glucose transporter receptors.

#### 5.2.2 Combining vaccines and metabolic targeting therapy

The combination of vaccines and metabolic targeting may synergistically benefit the treatment of PDAC by targeting metabolic pathways of immune cells to enhance their activity in the TME. This is additionally mediated by limiting nutrients in the TME to increase the number of activated immune cells and boost the immune response^182^. Studies have demonstrated that vaccines alone are not optimally effective in patients with PDAC, and adding metabolic targeting therapies to vaccines has given this immunotherapeutic modality a new promise and direction.

KIF20A, a kinesin superfamily protein 20A member, is an overexpressed motor protein in patients with PDAC. In a study enrolling patients with metastatic cancer who failed gemcitabine-based regimens, vaccination with a protein vaccine containing KIF20A-66 elicited a CD8^+^ effector T-cell response against the peptide. Moreover, the results revealed a median overall survival of 4.2 months^183^. The overexpression of vascular endothelial growth factor (VEGF) promotes the development and metastasis of PDAC as well as the appearance of aberrant vascular structures. In addition, patients with PDAC who co-expressed VEGF receptor 1/2 had a shorter survival. In a phase II/III clinical trial, the median overall survival rates of patients treated with combination of vascular endothelial growth factor receptor 2 (VEGF2) and peptide-based vaccine versus gemcitabine were comparable at 8.4 and 8.5 months, respectively^184^. Survivin 2B peptide (SVN-2B) is an essential protein that participates in programmed cell death and cell cycle regulation during embryonic and fetal development. SVN-2B activation is associated with drug resistance and poor prognosis in approximately 80% of patients with PDAC. Shima et al. developed a vaccine against SVN-2B to target survivin and enhance the number of CD8^+^ T cells specifically targeting survivin. Another protein is MUC1 which regulates signaling and cell differentiation in pancreatic tissues, is overexpressed in approximately 60% of patients with PDAC, and is strongly linked to PDAC development. A dendritic cell vaccine administered with MUC1-loaded MUC1 is non-toxic and can induce an immune response against the tumor antigen MUC1 in patients with advanced cancer^40^. MSLN is highly expressed in most patients with PDAC and is frequently activated alongside CA-125. The co-expression of both these genes promotes tumor invasion and metastasis. CRS-207 is a vaccine that has been demonstrated to enhance the frequency of MSLN-specific CD8^+^ T cells in 60% of patients with chemoresistant PDAC^185^. Understanding the mechanisms of PDAC resistance and underlying metabolic pathways in the TME may contribute to the development of more effective combination therapies to improve patient prognosis.

#### 5.2.3 Combining CAR-T with metabolic targeting therapy

Fusion of an antibody in a single-chain fragment variable with intracellular signaling structural domains such as CD3-chain, CD28, and CD137 generates CARs^186^. Specifically, CAR-T cells express CARs on their membranes, allowing them to recognize and attach to PDAC cells to produce specific antigens. CAR-T cells mediate MHC-unrestricted tumor cell killing by allowing T cells to bind to target cell surface antigens, which induces the apoptosis of PDAC cells^187^. Owing to the absence of co-stimulatory signaling, first-generation CAR-T cells with CD3 or FcR signaling structural domains have demonstrated low clinical efficacy. However, second- or third-generation CAR-T cells are more effective against PDAC with immunosuppressive TME. Reportedly, CAR-T cell therapy demonstrates excellent clinical efficacy in B-cell carcinoma; however, the optimal therapeutic combination for the treatment of this cancer type has not been determined. Nevertheless, it has been postulated that targeting PDAC metabolism may enhance CAR-T efficacy.

MSLN-targeting CAR-T cells may be effective against PDAC. A phase I clinical trial has demonstrated that treatment of a PDAC patient with MSLN-CAR-T cells resulted in subsequent stabilization, with no damage to the normal tissues. In addition, Beatty et al. found that two of six patients with PDAC who were treated with messenger ribonucleic acid-based MSLN-CAR-T cells had stable disease. Moreover, they reported that the metabolically active volume of individual tumor lesions remained stable in three patients and decreased by 69.2% in one patient with MSLN expression, as confirmed by biopsy^188^. In animal models, CAR-T cells targeting PSCA have demonstrated efficacy in PDAC cells. Furthermore, additional preclinical studies assessing the efficacy of CAR-T cells targeting upregulated MUC1 have demonstrated promising therapeutic effects. In another study, CAR-T was demonstrated to recognize the rare glycol-Tn antigen on MUC1 and thus mediate an excellent antitumor activity in a PDAC-loaded murine tumor model. Several early clinical trials are ongoing to evaluate the efficacy of MUC1-targeting CAR-T cells for the treatment of PDAC^189^.

### 5.3 Possibility of immunotherapy and RT in conjunction with metabolic targeting therapy

In recent times, RT has been regarded as an effective treatment for inhibiting the progression of numerous solid tumors^190^; however, for the treatment of PDAC, RT alone is ineffective. Among the innumerable molecular pathways via which RT mediates its efficacy, metabolic alterations in PDAC are a significant factor contributing to RT resistance^191^. This is because PDAC cells resist the effect of RT by inducing metabolic alterations. To this extent, clinical investigations have shown that RT is less effective in PDAC patients with high baseline metabolic levels^192^, ^193^. Specifically, MUC1 expression reduces RT-induced cytotoxicity and DNA damage in PDAC cells by upregulating glycolysis, the pentose phosphate pathway, and nucleotide biosynthesis^123^. However, a study has found that 2-deoxy-D-glucose, a glucose analog that competitively inhibits metabolism, induces a significant increase in metabolic oxidative stress by increasing glutathione disulfide accumulation and the NADP(+)/NADP(-) ratio, resulting in radiosensitization^194^. In mice with xenografts of MiaPaCa-2 cells, a high-fat, low-carbohydrate, ketogenic diet increased RT sensitivity. However, the extrapolation of this study to a phase I trial involving patients with PDAC did not produce the expected results owing to inadequate adherence (NCT01419483) 183. Moreover, studies have reported that fatty acid synthase upregulation causes RT resistance^195^, in addition to its effects on glucose metabolism. Notably, specific genes in the cholesterol synthesis pathway also contribute in some capacity to RT resistance. By inhibiting the overexpression of farnesyl diphosphate synthase (FDPS), zoledronic acid (Zol) can sensitize PDAC cells to RT^195^. In addition, Parthasarathy et al. have found that the overexpression of FDPS in PDAC cells was associated with a reduced RT response and decreased survival. In *in vitro* and *in vivo* studies, pharmacological inhibition of CRISPR/Cas9 and FDPS in human and mouse homozygous PDAC cells using Zol + RT increased PDAC radiosensitization. Intriguingly, mouse and human tumors treated with Zol + RT demonstrated substantial growth suppression. Reportedly, Zol induces radiosensitization by modulating Rac1 and Rho-organization, inducing DNA damage, altering radiation response signaling, and enhancing systemic immune cell activation. Preliminary findings of an ongoing phase I/II trial (NCT03073785) demonstrated that Zol + RT treatment improved failure-free survival, increased immune cell activation, and decreased microenvironment-associated transcripts. Overall, Zol improved the efficacy of RT by inhibiting FDPS, indicating a novel therapeutic mode of targeted metabolism^196^.

PDAC is characterized by minimal immunogenicity and a highly immunosuppressive microenvironment. Specifically, the role of TME is mediated by three major immune cell types: Tregs, M2-type TAMs, and MDSCs. In PDAC, immunosuppression and immunotherapeutic resistance are closely linked with metabolism. Aerobic glycolysis plays an important role in activating immune function by CD8^+^ effector T cells and producing interferons by CD4^+^ T cells^197^, ^198^. In contrast, glycolysis is critical for the functioning of CD4^+^ effector T cells (Th1, Th2, and Th17) and M1 TAMs. Tregs and M2-type TAMs are chiefly fueled by lipid oxidation^199^, ^200^. Notably, tumor-associated MDSCs undergo metabolic reprogramming to sustain survival and effectuate their immunosuppressive functions via increased FAO and glycolysis^201^, ^202^. In addition, tumor-derived lactate elicits an M2-like phenotype and downregulates the function of CD8^+^ cytotoxic T cells in TAMs, further remodeling the TME^203^, ^204^. Importantly, lactate induces an immunosuppressive microenvironment in PDAC by upregulating MDSC and inhibiting natural killer cell activity^205^. In addition, in a mouse model of caloric restriction, tumor-induced IL-6 was found to compromise its metabolism (ketogenic response), thus suppressing anti-tumor immunity^206^. In conclusion, targeted metabolic therapies offer novel approaches to immunosuppression and contribute to novel concepts for immunotherapy combination therapies.

Owing to the poor prognosis of surgical treatment for PDAC and the poor tolerability of RT and chemotherapy, immunotherapy has emerged as an alternative modality for the treatment of PDAC in recent years. Several immunotherapies for PDAC, including ICIs^207^ and therapeutic vaccines^208^, ^209^, are being evaluated in clinical trials; however, most immunotherapies alone have suboptimal outcomes. Recent research indicates that immunotherapy mediated by T cells can optimize treatment efficacy by modulating cellular metabolism^210^. In addition, ICIs can support lymphocyte metabolism in tumors and enhance their anti-tumor effects^124^, ^211^. Given the relationship between immunotherapy and metabolism, it is possible to integrate metabolic modulation with conventional immunotherapy. In addition to enhancing the capacity of lymphocytes to combat tumor effects, targeting the immunosuppressive TME is emerging as a new therapeutic option^212^. Several cytokines also play a significant role in PDAC immunosuppression. IDO, a metabolic enzyme expressed in most human tumors, inhibits immune cell responses by metabolizing tryptophan in the TME^213^, ^214^. Thus, by combining the inhibition IDO and of tumor connective tissue proliferation, Edwin et al.^215^ achieved significant anti-tumor activity in a mouse model of PDAC. An ongoing phase II trial (NCT03006302) is evaluating the efficacy of IDO inhibitors and immunotherapy or cyclophosphamide for the treatment of patients with PDAC. Given the significance of metabolism in immunity, metabolic modulation has the potential to enhance the clinical efficacy of immunotherapies.

## 6. Conclusion

PDAC is an aggressive malignancy and is associated with one of the highest mortality rates among all cancers. There have not been considerable advances in PDAC treatment owing to the immunosuppressive TME of PDAC. Although immunotherapy has demonstrated improved efficacy for treating several cancers, ICIs and vaccines are less effective in patients with PDAC. This failure of immunotherapy is primarily attributable to the immunosuppressive microenvironment of PDAC. First, the dense stroma of PDAC TME acts as a physical barrier to immunotherapeutic drug delivery. By converting to CAF, astrocytes in the TME induce collagen deposition and promote fibrosis. Second, the immunosuppressive TME of PDAC encourages the proliferation of immunosuppressive cells, the suppression of immune effector cells, and a reduction in antigen presentation. Moreover, the immunosuppressive characteristics of the TME substantially contribute to the production of metabolites, receptors, and cytokines. RT has demonstrated an immunomodulatory effect, which can help avoid immunotherapeutic resistance to PDAC-antigen presentation and T-cell activation. Although RT plays a significant role in improving immunotherapy efficacy in patients with PDAC, targeting the TME is more of an “on/off” control that determines the overall efficacy of PDAC therapy. In patients with PDAC, neither RT alone nor RT in combination with immunotherapy has been demonstrated to be effective. Therefore, it is critical to determine effective methods to modulate the complex interactions in the TME for the development of PDAC therapeutic strategies. In cancer, metabolic reprogramming is a convergence of biological phenomena and potential clinical targets. Despite the discovery of several potential metabolic molecules, clinical trials focusing on the metabolism of cancer therapies have not yielded significant results. Several factors, including adverse effects, metabolic heterogeneity, flexibility and plasticity, and complex interactions between tumors and the TME limit the efficacy of metabolic therapies. To overcome these obstacles, numerous steps must be taken. Integration of multi-omics, single-cell, and spatial assays is required to identify more preferable metabolic targets. In addition, the development of metabolomics and other technologies could expedite the discovery of novel metabolic targets and pathways. The development of precision medicine approaches in combination with metabolically targeted therapies is another crucial area of research. These approaches consider an individual's metabolic profile, resulting in the development of more personalized therapies. However, integrated computational modeling and experimental methodologies are essential for the advancement of these precision medicine approaches. In addition, tools to monitor and visualize metabolic reorganization processes more precisely and dynamically are required. These developments will help with advancements in personalized metabolic therapies for patients in clinical settings. The metabolic targets shared by PDAC cells and immune cells require further investigation to develop new therapeutics and treatment methods for PDAC. However, a few key questions that need to be addressed for combination immunotherapy remain: how to identify the unique metabolic pathways and metabolites of PDAC cells as targets for targeted inhibition, how to utilize the plasticity of differential metabolism, and how to balance tumor suppression and maintenance of immune cell activity.

## Figures and Tables

**Table 1:** Status of the combination of immunotherapy and RT in clinical research

Systemic agents	Phase	N	RT scheme	Note	Reference
Gemcitabine + pembrolizumab + trametinib	II	147	35 to 40 Gy	mOS: 15.1 months	168
Durvalumab + tremelimumab	I	59	1 × 8 Gy or 5 × 5 Gy	ORR: 5.1%	169
Nivolumab + ipilimumab	II	25	3 × 8 Gy	ORR: 12%	170
Pembrolizumab + trametinib	II	85	5 × 7-8 Gy	mOS: 14.9 months	171
Nivolumab +/-ipilimumab	II	84	1 × 15 Gy	mOS: 3.8 months ORR: 14% for the triple combination	175
Gemcitabine + algenpantucel-L (cancer vaccine)	II	70	28 × 1.8 Gy (+ 5-FU)	1-year DFS: 62%	176
FFX or Gem-Np +/- algenpantucel-L	III	303	28 × 1.8 Gy (+ 5-FU or capecitabine)	mOS: 14.3 months (vs. 14.9 for SOC)	177
GVAX (cancer vaccine)	II	60	28 × 1.8 Gy (+ 5-FU)	mDFS: 17.3 months	178

RT: radiotherapy; ORR: objective response rate; mOS: median overall survival.

## Abbreviations

PDAC: pancreatic ductal adenocarcinoma
RT: radiotherapy
TME: tumor microenvironment
ICIs: immune checkpoint inhibitors
PD-1: programmed cell death protein-1
PD-L1: programmed cell necrosis protein ligand-1
CTLA-4: cytotoxic T-lymphocyte-associated antigen 4
CAFs: cancer-associated fibroblasts
Tregs: regulatory T cells
TAMs: tumor-associated macrophages
MDSCs: myeloid-derived suppressor cells
NETs: neutrophils
IL-10: interleukin 10
TGF-β: transforming growth factor-β
OXPHOS: oxidative phosphorylation
MSI-H: high satellite instability
ORR: objective response rate
MUC1: mucin 1
GM-CSF: granulocyte-macrophage colony-stimulating factor
GVAX: GM-CSF gene transduced autologous tumor vaccine
CSF-1R: colony-stimulating factor 1-receptor
CAR-T: chimeric antigen receptor T-cell
DCs: dendritic cells
CEA: carcinoembryonic antigen
SBRT: stereotactic body RT
TLS: tertiary lymphoid structures
CCL2: chemokine Ligand 2
M-CSF: macrophage colony-stimulating factor
G-MDSC: granulocyte MDSC
ROS: reactive oxygen species
Cx43: connexin-43
GPR81: g-protein-coupled receptor 81
FAO: fatty acid oxidation
VLCAD: very-long-chain acyl-CoA dehydrogenase
LCFAs: long-chain fatty acids
NADPH: nicotinamide adenine dinucleotide phosphate
ATM: ataxia telangiectasia-mutated gene
SHP-1: src homology region 2 domain-containing phosphatase-1
SHP-2: src homology region 2 domain-containing phosphatase-2
VEGF: vascular endothelial growth factor
VEGF2: vascular endothelial growth factor receptor 2
SVN-2B: survivin 2B peptide
FDPS: farnesyl diphosphate synthase
Zol: zoledronic acid
IDO: indoleamine 2,3-dioxygenase
ORR: objective response rate
mOS: median overall survival
CTLs: cytotoxic lymphocytes
CXCL12: c-x-c motif chemokine ligand 12
FAK: focal adhesion kinase
